# Automated Aircraft Dent Inspection via a Modified Fourier Transform Profilometry Algorithm

**DOI:** 10.3390/s22020433

**Published:** 2022-01-07

**Authors:** Pasquale Lafiosca, Ip-Shing Fan, Nicolas P. Avdelidis

**Affiliations:** Integrated Vehicle Health Management Centre, Cranfield University, Cranfield MK43 0AL, UK; i.s.fan@cranfield.ac.uk (I.-S.F.); np.avdel@cranfield.ac.uk (N.P.A.)

**Keywords:** airworthiness, fringe patterns, pinhole camera model, structured light, inspections

## Abstract

The search for dents is a consistent part of the aircraft inspection workload. The engineer is required to find, measure, and report each dent over the aircraft skin. This process is not only hazardous, but also extremely subject to human factors and environmental conditions. This study discusses the feasibility of automated dent scanning via a single-shot triangular stereo Fourier transform algorithm, designed to be compatible with the use of an unmanned aerial vehicle. The original algorithm is modified introducing two main contributions. First, the automatic estimation of the pass-band filter removes the user interaction in the phase filtering process. Secondly, the employment of a virtual reference plane reduces unwrapping errors, leading to improved accuracy independently of the chosen unwrapping algorithm. Static experiments reached a mean absolute error of ∼0.1 mm at a distance of 60 cm, while dynamic experiments showed ∼0.3 mm at a distance of 120 cm. On average, the mean absolute error decreased by ∼34%, proving the validity of the proposed single-shot 3D reconstruction algorithm and suggesting its applicability for future automated dent inspections.

## 1. Introduction

Maintenance, repair and overhaul (MRO) companies have an interest in the progressive automation of aircraft inspections. These are not only hazardous, but also costly, time-consuming, labour-intensive, and subject to human error [1], yet are critical for the *airworthiness* assessment. The potential advantages of automation are evident: improved safety, higher productivity, reduced aircraft downtime, and standard output. Therefore, the general problem of automating inspections has been attracting the attention of many researchers lately, targeting damages on aircraft *skin* for its extent and its desirable characteristic of being directly accessible. Understandably, unmanned aerial vehicles (UAVs) are often considered to reach areas of the skin at a certain height, relieving the engineer from connected safety risks [2]: in 2018, Airbus launched an indoor inspection drone [3] and recently approved UAVs for lightning checks [4]. Similarly, other companies, such as easyJet and Air France Industries-KLM, are pioneering the adoption of UAVs for inspections.

Among inspection technologies that enable the use of UAVs [5], pattern recognition is at the basis of several commercial products recently introduced in MRO. It has the advantage of being easy to implement, not requiring particular hardware except for a high-definition camera, and has been successfully applied for detecting scratches and lightning damage. Thermal imaging has also been proposed for its capability to detect delamination and corrosion [6]. Despite their relevance and frequency of occurrence on the aircraft skin, little has been carried out for dents. Maintenance procedures require periodic and comprehensive inspections during which dents must be found, measured, and reported. However, automation of dent inspections poses new challenges due to the nature of this damage.

A dent is smooth and without well-defined boundaries, with depth easily lower than 1 mm. As the aircraft skin is generally single-coloured and lacking in texture, a dent may not be clearly distinguishable even by trained engineers, who usually rely on reflections or on the light of a torch that seeps under a ruler placed on the surface, to highlight depth variations and spot dent locations during general visual inspections. Furthermore, *measures* of depth and width are required to classify a dent as *allowable damage* or not [7]. In traditional inspections, measures are collected by means of a depth gauge and a ruler. Although high-accuracy handheld 3D scanning tools are available, quick but less-accurate traditional methods still account for the most part [8,9] and 3D scanners are reserved for special inspections. The reason is probably to be found in the relatively short operating distances of these devices and in the fact that the aircraft skin includes locations at height normally reached by platforms and climbing equipment. As such, handheld scanning devices not only still require the engineer to reach those locations, they also add practical difficulties for carrying them and increased setup time and scan data analysis effort.

Among the few works addressing dent inspections, Jovancevic et al. [10] proposed the use of Air-Cobot equipped with commercial 3D scanners and a region-growing algorithm for the automatic segmentation of dented areas. However, it left open the problem of reaching the upper part of the aircraft. Doğru et al. [11] proposed a convolutional neural network to detect dents, suggesting the use of UAVs to achieve full automation. Still, the use of monocular images not only prevents measuring the damage but also results in low accuracy. The capability to detect a dent with this method is virtually zero for shallow dents over nontextured skin, which are the most common and nevertheless must be found and reported. Hence, the acquisition of 3D data cannot be avoided.

In the abovementioned context, this article discusses the feasibility of automated dent scanning via a single-shot structured-light 3D scanner. With respect to multiple-shot, the choice of a single-shot algorithm makes the system resilient to fast movements and vibration during scanning. With further development, such a system could be integrated on a UAV, enabling quick and effective automated dent inspections. After a brief review of the available structured-light codification strategies, an algorithm based on the Fourier transform profilometry (FTP) is tested for the scope. The use of a *virtual* reference plane is proposed for:

- The automatic band-pass estimation (Section 2.2), thus eliminating the user interaction in the filtering process.
- The reduction of errors in the phase unwrapping process via a virtual reference image (Section 2.4).

Simulations (Section 3) and experiments (Section 4) on both static and moving surfaces presenting artificial dents prove the validity of the proposed algorithm and show that the use of FTP is promising for the automation of aircraft dent inspections. Discussion and Conclusions follow in Section 5 and Section 6.

### Structured-Light Codification Strategies

Considering the purpose of this work, only the codification strategies applicable to a minimal structured-light system, composed of a camera and a projector, are taken into account. Therefore, strategies involving the use of multiple cameras, additional constraints in the system geometry, or restricted depth range are omitted.

Coding approaches can usually be classified as time-multiplexing or spatial neighbourhood. Time-multiplexing (or *multiple-shot*) is capable of obtaining higher resolution and accuracy. Techniques in this group can be roughly divided into Gray coding and temporal phase shifting, or a combination of the two [12]. The main advantage of phase-based coding over Gray coding is to provide sub-pixel accuracy, thanks to the use of continuous functions. The large number of projected patterns makes time-multiplexing unsuitable for moving objects [13]. Methods using a limited number of patterns (≥2) and high-speed devices can be found in the literature [14,15,16]; however, their employment on moving objects, outside laboratory conditions, and without high-end hardware is still an active field of research.

Spatial neighbourhood (or *single-shot*) enables the measurement of moving surfaces and is insensitive to vibrational noise [17]. This comes at the price of lower accuracy, as the coding information must be contained in one pattern only, and local smoothness must be assumed for the neighbourhood to be correctly decoded. Spatial methods can use colour or greyscale values. In colour-based approaches, a compromise is to be found between the number of colours and noise sensitivity, while the use of greyscale intensity values results in more robustness [13]. Similarly to time-multiplexing, spatial coding can also be divided into discrete and continuous. The De Bruijn colour sequence [18] is probably the most representative discrete method, from which several techniques have followed [19,20,21]. Discrete methods extract *codewords* from local areas, which in most cases require several pixels, limiting resolution. Continuous spatial coding, instead, uses a smooth pattern to obtain a *dense* surface reconstruction.

Among the latter, Fourier transform profilometry (FTP) aims at calculating the continuous phase value from a single projected pattern through a filter in the frequency domain [22,23]. The filtering is a critical step, and aliasing can prevent the extraction of the fundamental spectrum. It also poses a limit to the phase derivative along the fringe direction, but not directly to the maximum measurable height [22]. With nonstationary signals, the classic FTP may have difficulties in isolating the first harmonic. In order to solve this, windowed FTP and wavelet transform provide a space–frequency representation demanding high computational cost [17,24,25,26] and the choice of additional system parameters, although approaches for the automatic selection of the window size have been proposed [27,28].

Finally, in both time-multiplexing approaches and FTP, the phase is used to calculate the object 3D coordinates. However, while time-multiplexing is generally able to obtain the absolute phase directly (via *temporal* unwrapping), FTP can only provide a *wrapped* phase that needs to be processed through a *spatial unwrapping* algorithm and offset to obtain the absolute phase, not without difficulties [29,30]. The absolute phase can then be used to find a correspondence between camera and projector, and thus proceed with stereo triangulation [31,32], or the depth can be seen as a function of the phase difference from a physical reference plane [22,30,33].

## 2. Proposed Method

In this study, a modified triangular stereo FTP method is implemented to discuss its compatibility and performance towards automated dent inspections. In addition, two contributions are presented.

First, the automatic estimation of the band-pass filter is proposed (Section 2.2). This feature is essential, as the first-spectrum band varies with the distance from the scanned surface and the system should be able to proceed *without user interaction*.

Secondly, a strategy for the reduction of phase unwrapping errors is shown (Section 2.4). All phase-based single-shot methods initially output a wrapped (or modulo 2π) phase. After spatial unwrapping, two methods can be found in the literature to relate phase with depth. In systems calibrated using the pinhole model [34], the object phase can be directly used to solve the stereo correspondence problem, thus proceeding with triangulation [31,32]. Alternatively, the depth can be seen as a function of the *phase shift* of the object, calculated with respect to a *reference plane*, as commonly performed in phase-height mapping methods [22,33]. While triangular stereo methods are generally more accurate, suitable for extended depth range measurements, and easier to calibrate out of the lab [30,35], here, it is shown that the employment of the phase shift has the benefit of reducing unwrapping errors and that its advantage can be brought to triangular stereo methods by means of a *virtual* reference plane.

### 2.1. Notation and System Geometry

**Figure 1:**
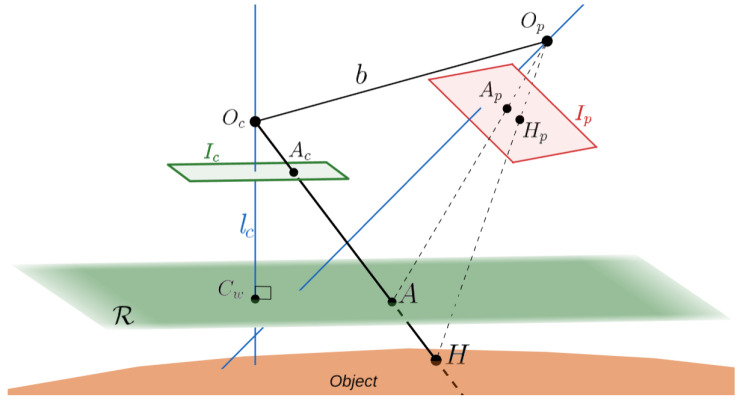
System configuration: camera and projector are freely positioned (optical axes in blue solid). Without loss of generality, camera is placed in the world origin and a virtual reference plane R lays in front of the camera.

Throughout the article, the pinhole camera model is used for both camera and projector [34]. Without loss of generality, the camera optical centre is placed in the world origin $O_{c}=\left(0,0,0\right)$, oriented in the same way so that the world *z*-axis corresponds to the camera optical axis. $K_{c}$ is the camera intrinsic matrix, while its rotation matrix and translation vector, due to its position, are simply $R_{c}=I$ (3×3 identity matrix) and $t_{c}=0$, respectively. The projector optical center is placed in $O_{p}=\left(x_{p},y_{p},z_{p}\right)$, freely oriented. Its intrinsic matrix, rotation matrix, and translation vector are identified by $K_{p}$, $R_{p}$, and $t_{p}$, respectively. Lens distortion correction is managed using the radial and tangential model [34], but is not explicitly reported in the following formulae. Camera-projector calibration is assumed as known, following one of the several methods available in the literature [31,36,37,38]. The described system geometry is represented in Figure 1.

$I_{c}$ and $I_{p}$ are the camera and projector image planes, respectively. In particular, $I_{c}$ is the plane where the image acquired by the camera lays *after* distortion correction has been applied, while the distortion-free fringe image chosen as projector input lays on $I_{p}$. On each image, a pixel coordinate system is defined, with $\left(u_{c},v_{c}\right)\in I_{c}$ and $\left(u_{p},v_{p}\right)\in I_{p}$ being two generic points laying on camera and projector images, respectively.

**Figure 2:**
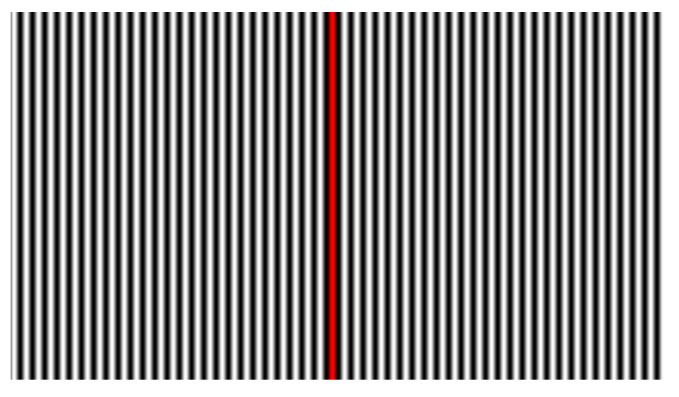
A fringe pattern with period $T_{p}$, with a central red-coloured stripe.

A sinusoidal fringe pattern is chosen with fringes perpendicular to the $u_{p}$-axis, having period $T_{p}$ (in pixels) and frequency $f_{p}=1/T_{p}$. A red-coloured stripe is drawn for one central period, following the intensity trend (Figure 2). After projection, the corresponding points can be identified from the camera using a weighted average of red intensity values over the *u*-axis, producing sub-pixel accuracy. The use of a similar centerline was also proposed in [31].

The equation of the virtual reference plane R is considered at $z=l_{c}$, thus laying perfectly in front of the camera, where $l_{c}$ is chosen as the average depth of the red stripe world points, readily calculated by triangulation.

The aim of FTP is to find the generic 3D point *H* belonging to the object, observed as $A_{c}\in I_{c}$ and $H_{p}\in I_{p}$, with *A* being the intersection of the ray $\bar{O_{c}H}$ and R.

### 2.2. Automatic Band-Pass Filter

In FTP, a band-pass filter must be applied to isolate the fundamental spectrum and filter out DC component and upper harmonics [17,22,29]. The band of interest can be identified by the interval $\left[f_{c}-r,f_{c}+r\right]$, where $f_{c}$ is the fundamental frequency, as seen from the camera, and *r* is an appropriate radius. Experimentally, it can be shown that the fundamental spectrum varies significantly when scanning objects at different distances, requiring the human interaction for the manual selection of the band-pass at each scan. This becomes impractical when dealing with a large number of scans with varying distances. The automatic band-pass filter solves this problem.

The estimation of $f_{c}$ assumes that the area of major interest is at depth $z=l_{c}$ (as calculated in Section 2.1). The intersection of camera optical axis and R is simply $C_{w}={\left(0,0,l_{c}\right)}^{T}$, that is projected on the projector image plane to find $C_{p}=\left(u_{p},v_{p}\right)\in I_{p}$:

(1) $$\left(\begin{array}{c} u_{p}\\ v_{p}\\ 1 \end{array}\right)=\mathrm{Normalise}\left(K_{p}\;\cdot \;\left[R_{p}|t_{p}\right]\left(\begin{array}{c} 0\\ 0\\ l_{c} \end{array}\right)\right)$$

where Normalise() is a function that divides a homogeneous coordinate by its 3^rd^ element. On $I_{p}$, two points can be identified as

(2) $$C_{p}^{left}=\left(\begin{array}{c} u_{p}-\frac{T_{p}}{2}\\ v_{p}\\ 1 \end{array}\right)\;C_{p}^{right}=\left(\begin{array}{c} u_{p}+\frac{T_{p}}{2}\\ v_{p}\\ 1 \end{array}\right)$$

covering a full period $T_{p}$. Then, $C_{p}^{left}$ is deprojected back to R and, from here, to the camera:

(3) $$\begin{array}{cc} C_{c}^{left}=\; & \mathrm{Normalise}(K_{c}\;\cdot \;M\;\cdot \;\\ \; & \mathrm{Normalise}({\left(K_{p}\;\cdot \;R_{p}\right)}^{-1}\;\cdot \;\left(\begin{array}{c} u_{p}-\frac{T_{p}}{2}\\ v_{p}\\ 1 \end{array}\right))) \end{array}$$

where:

(4) $$M=\left(\begin{array}{ccc} l_{c}-z_{p} & 0 & x_{p}\\ 0 & l_{c}-z_{p} & y_{p}\\ 0 & 0 & l_{c} \end{array}\right)$$

The same applies to $C_{p}^{right}$, giving $C_{c}^{right}$. Lens distortion is corrected accordingly in all of the steps. $C_{c}^{left}$ and $C_{c}^{right}$ lie on $I_{c}$ at a distance corresponding to the *projector* period and its orientation. From Pythagorean geometry, $T_{c}$ is calculated as the projection of $\bar{C_{c}^{left}C_{c}^{right}}$ over the $u_{c}$-axis. Finally, $f_{c}=1/T_{c}$ and, throughout this paper, $r=f_{c}/2$.

**Figure 3:**
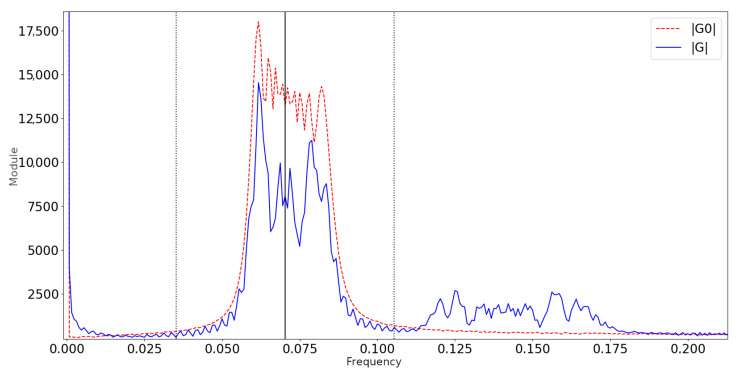
Module of the FFT on both reference (dotted red) and object (solid blue) images along middle row for a real dent sample. Spectrum around the fundamental frequency (solid vertical) is isolated with the automatic band-pass filter (dotted vertical).

The band-pass estimation does not require any user input and allows for some automation [29,35] and extended adaptability of FTP in different system geometries and at varying distances from the object, particularly when scanning surfaces roughly placed in front of the camera, as in the application here discussed. The automatic band-pass can successfully isolate the main spectrum (Figure 3) and it was successfully used in all the following simulations (Section 3) and experiments (Section 4).

### 2.3. Virtual Reference Image

On the domain of the points of R observed from the camera and illuminated by the projector, the relation between projector and camera image planes is *bijective*. This allows to virtually build a reference image as seen from the camera.

The generic point $A_{p}=\left(u_{p}^{A},v_{p}^{A}\right)\in I_{p}$ is projected to the world point A∈R, which is, in turn, observed from the camera as $A_{c}=\left(u_{c}^{A},v_{c}^{A}\right)\in I_{c}$. $A_{p}$ is calculated from $A_{c}$ as

(5) $$\left(\begin{array}{c} u_{p}^{A}\\ v_{p}^{A}\\ 1 \end{array}\right)=\mathrm{Normalise}\left(K_{p}\;\cdot \;\left[l_{c}R_{p}K_{c}^{-1}|t_{p}\right]\left(\begin{array}{c} u_{c}^{A}\\ v_{c}^{A}\\ 1\\ 1 \end{array}\right)\right)$$

In other words, the effect of Equation (5) is to find the line passing through $O_{c}$ and $A_{c}$ that intersects R at a certain world point *A*, and then project *A* onto $I_{p}$. As usual, camera and projector coordinates are also corrected for lens distortion.

With this simple strategy, each point of the camera image is mapped to its corresponding point and its intensity value on the projected fringe image on $I_{p}$. As the mapping generally produces noninteger numbers, the final camera image is derived with cubic interpolation. The red stripe is not needed in this image.

**Figure 4:**
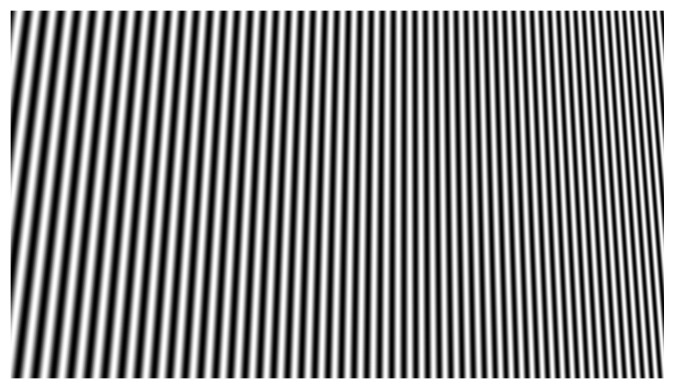
Virtually built reference image with sinusoidal fringes. Perspective affects frequency components.

The phase variation due to perspective is clearly visible in Figure 4. As for its frequency content, the virtual reference image is the same as the one that would be acquired placing a real plane at the same position. However, the effects of light diffusion, object albedo, and environment illumination are not introduced.

The virtual reference image is then used to calculate the phase shift, similarly to phase-height methods [22,33] but without a physical plane. The usefulness of this is explained below.

### 2.4. Reduction of Noise Effect over Phase Jumps

With single-shot FTP, the choice of unwrapping algorithms is limited to the spatial ones. To calculate the absolute phase Φ, estimated as $\tilde{\Phi }$, spatial phase unwrapping cannot rely on information other than the phase map $\Phi^{w}$ that, due to the presence of noise, may present *genuine* or *fake* 2π-jumps [39]. The two are indistinguishable, making the spatial unwrapping such a challenging task [40].

In both stereo triangulation and phase-height mapping methods, $\tilde{\Phi }$ is related to the world *z* coordinate. While stereo triangulation directly relates $\tilde{\Phi }$ to a point on $I_{p}$ [31,32], phase-height mapping estimates *z* as a function of the *phase shift* $\Delta \tilde{\Phi }$ from a reference phase $\Phi_{0}$ calculated over a plane [22,33].

Although the proposed method uses a stereo triangulation approach, it also exploits the calculation of $\Delta \tilde{\Phi }$ with respect to a *virtual* reference image (Section 2.3) to reduce the number of 2π-jumps, both genuine and fake ones. Consequently, unwrapping errors are reduced, as there is less probability for the noise to interfere with 2π-jumps.

The virtual reference and the object intensity values observed from the camera can be represented, respectively, as

(6) $$\begin{array}{cc} g_{0}\left(u_{c},v_{c}\right) & =A_{0}\left(1+\cos\Phi_{0}\right) \end{array}$$

(7) $$\begin{array}{cc} \; & =A_{0}\left\{1+\cos\left[2\pi f_{c}u_{c}+\varphi_{0}\left(u_{c},v_{c}\right)\right]\right\} \end{array}$$

and

(8) $$\begin{array}{cc} g(u_{c},v_{c}) & =A(1+\cos\Phi ) \end{array}$$

(9) $$\begin{array}{cc} \; & =A\{1+\cos\left[2\pi f_{c}u_{c}+\varphi \left(u_{c},v_{c}\right)\right]\} \end{array}$$

where $f_{c}$ is the fundamental frequency (Section 2.2), $A=A(u_{c},v_{c})$ and $A_{0}=A_{0}\left(u_{c},v_{c}\right)$ are intensity modulations, $\varphi_{0}\left(u_{c},v_{c}\right)$ represents the phase modulation over the virtual reference image (caused by perspective only), and $\varphi (u_{c},v_{c})$ is the phase modulation over the object caused by perspective, object depth, and noise.

After fast Fourier transform (FFT), filtering and inverse FFT (without aliasing) of *g*, the wrapped object phase can be obtained as

(10) $$\Phi^{w}\left(u_{c},v_{c}\right)=arg\left[\frac{A}{2}e^{i\Phi (u_{c},v_{c})}\right]=W\left[2\pi f_{c}u_{c}+\varphi \left(u_{c},v_{c}\right)\right]$$

where W[·] stands for the wrapping operation (or modulo 2π). Then, the classic direct method estimates the unwrapped object phase as

(11) $${\tilde{\Phi }}^{r}\left(u_{c},v_{c}\right)=U\left[\Phi^{w}\left(u_{c},v_{c}\right)\right]$$

where U[·] represents a generic spatial unwrapping algorithm. Note that ${\tilde{\Phi }}^{r}$ is still a *relative* phase, with uncertain fringe order.

Furthermore, as in phase-height mapping methods [22,33], the wrapped phase shift can be calculated from a new signal obtained as the filtered object signal multiplied by the complex conjugate of the filtered reference [22]:

(12) $$\begin{array}{cc} \Delta \Phi^{w}\left(u_{c},v_{c}\right) & =arg\left[\frac{A}{2}e^{i\Phi (u_{c},v_{c})}\;\cdot \;\frac{A_{0}}{2}e^{-i\Phi_{0}\left(u_{c},v_{c}\right)}\right]\\ & =W[\varphi \left(u_{c},v_{c}\right)-\varphi_{0}\left(u_{c},v_{c}\right)] \end{array}$$

and the unwrapped object phase can be obtained also, as

(13) $$\begin{array}{cc} {\tilde{\Phi }}^{r}\left(u_{c},v_{c}\right) & =\Phi_{0}\left(u_{c},v_{c}\right)+U\left[\Delta \Phi^{w}\left(u_{c},v_{c}\right)\right]\\ \; & =\Phi_{0}\left(u_{c},v_{c}\right)+\Delta {\tilde{\Phi }}^{r}\left(u_{c},v_{c}\right) \end{array}$$

where $\Phi_{0}\left(u_{c},v_{c}\right)$ is the absolute reference phase and $\Delta {\tilde{\Phi }}^{r}$ is the unwrapped phase shift.

**Figure 5:**
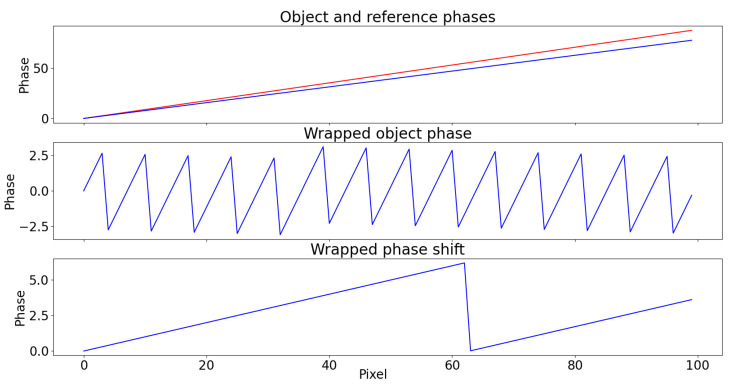
(**Top**) Illustrative reference $\Phi_{0}$ (blue) and object Φ (red) phases. (**Middle**) The wrapped object phase $\Phi^{w}$ presents many more 2π-jumps compared to the (**Bottom**) wrapped phase shift $\Delta \Phi^{w}$.

Equations (11) and (13) should lead to the same result. However, the interaction of noise over 2π-jumps will be greater in Equation (11) than in Equation (13). In fact, under the general assumption of FTP, φ and $\varphi_{0}$ must vary very slowly compared to the fundamental frequency $f_{c}$ [22], and as a direct consequence, $\Delta \Phi^{w}$ presents less 2π-jumps compared to $\Phi^{w}$. An example is shown in Figure 5.

Therefore, Equation (13) allows to deal with the unwrapping of the signal $\Delta \Phi^{w}$ that contains a slower trend superimposed modulo 2π on the same noise distribution found on $\Phi^{w}$ (which originates from the sole object image, as the virtual reference is noise-free). This leads to a more faithful unwrapped phase.

**Figure 6:**
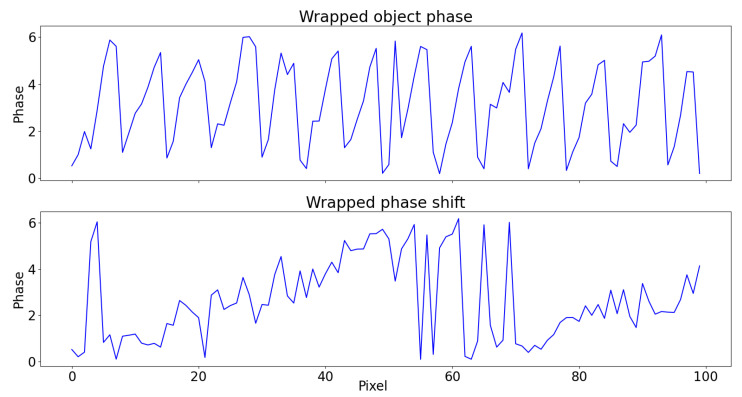
(**Top**) The wrapped object phase $\Phi^{w}$ and (**Bottom**) wrapped phase shift $\Delta \Phi^{w}$ with the same white noise (σ=0.6) added.

To show the practical effect of this, a white noise (σ=0.6) is added to Φ of the same example of Figure 5. The wrapped object phase and phase shift are shown in Figure 6: it is clear that the task of unwrapping becomes more challenging due to noise interaction with jumps and that the probability for it to interfere are lower in the second case, due to fewer 2π-jumps.

**Figure 7:**
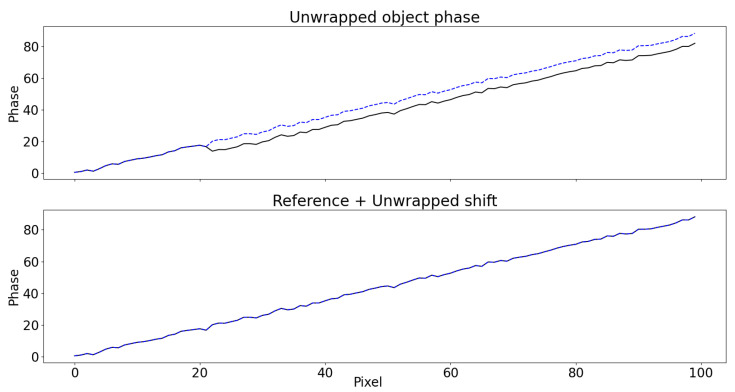
The unwrapped object phase ${\tilde{\Phi }}^{r}\left(u_{c},v_{c}\right)$ using (**top**) Equation (11) and (**bottom**) Equation (13) with respect to the original phase (dashed blue). In the first case, the noise causes the unwrapping algorithm to discard a genuine jump right after $u_{c}=20$, introducing error. This does not happen in the second case due to the reduced presence of 2π-jumps.

The unwrapped phase using the algorithm by Itoh [41] is shown in Figure 7. Even in this very simple case, noise causes a genuine jump right after $u_{c}=20$ to be missed, thus propagating the error to the right. The unwrapping of $\Delta \Phi^{w}$ in Equation (13), instead, is more robust and outputs the correct result.

Basic single-shot structured-light applications cannot rely on additional knowledge to eliminate noise, and a spatial unwrapping algorithm cannot count on information other than the wrapped phase itself. Therefore, the reduction of 2π-jumps increases performance, regardless of the unwrapping algorithm used. This was confirmed by the following simulations (Section 3) and experiments (Section 4).

Furthermore, simulations in the absence of noise revealed that the use of Equation (13) in place of Equation (11) increases the numerical precision. This can be explained by the fact that, in computers, complex numbers are generally stored in algebraic form and the argument is extracted by means of the arctan2 function. Numerically, its asymptote regions are more sensitive to machine precision error, and Equation (13) leads to reduced error, as the slow-growing signal crosses those regions fewer times. Depending on sampling and period, the error $∥\tilde{\Phi }-\Phi ∥$ decreased up to $10^{-6}$.

### 2.5. Stereo Correspondence

Before proceeding with triangulation, the relative unwrapped phase ${\tilde{\Phi }}^{r}$ must be shifted accordingly to obtain the absolute phase $\tilde{\Phi }$, following

(14) $$\tilde{\Phi }={\tilde{\Phi }}^{r}+2k\pi$$

where k∈K is the difference from the correct fringe order [40].

Additional information is needed to find $\tilde{\Phi }$, for example, the absolute phase value in correspondence of the red stripe [31]:

(15) $$\tilde{\Phi }\left(u_{c},v_{c}\right)={\tilde{\Phi }}^{r}\left(u_{c},v_{c}\right)-\frac{\sum_{n=0}^{N}{\tilde{\Phi }}_{n}\left(u_{c},v_{c}\right)}{N}$$

where *N* is the number of pixels of the red stripe and ${\tilde{\Phi }}_{n}\left(u_{c},v_{c}\right)$ phase values at stripe locations. Then, each $\tilde{\Phi }\left(u_{c},v_{c}\right)$ value may be directly associated with its $u_{p}$ coordinate, as

(16) $$u_{p}=\frac{W_{p}\;\cdot \;\tilde{\Phi }\left(u_{c},v_{c}\right)}{2\pi }$$

where $W_{p}$ is the projector resolution along the $u_{p}$-axis.

Equivalently, Equation (14) can be rewritten as

(17) $$2\pi f_{p}u_{p}=2\pi f_{p}u_{p}^{A}+\Delta {\tilde{\Phi }}^{r}\left(u_{c}^{A},v_{c}^{A}\right)+2k\pi$$

where $u_{p}^{A}$ is obtained through Equation (5). Applying the above relation for each of the red stripe *u*-coordinates found on $I_{c}$ allows to resolve for *k*. The average *k* is selected and rounded to the nearest integer. Numerically, the latter approach delivers increased accuracy, as *k* is correctly forced to be an integer.

Once *k* is known, Equation (17) can be used to retrieve all the $u_{p}$ points with respect to each camera point. Finally, $v_{p}$ is calculated via epipolar geometry, and having all the corresponding couples, $\left(u_{c},v_{c}\right)\in I_{c}$ and $\left(u_{p},v_{p}\right)\in I_{p}$, one may proceed with stereo triangulation.

## 3. Computer Simulations

In the aircraft structural repair manual, a dent is defined as a damaged area pushed from its normal contour that presents smooth edges [7]. The length of a dent is the longest distance from one end to the other, while the width is the maximum distance measured at $90^{\circ }$ from the direction of the length. The depth is measured at their intersection. A dent is usually classified by its width, depth, and width/depth ratio.

**Figure 8:**
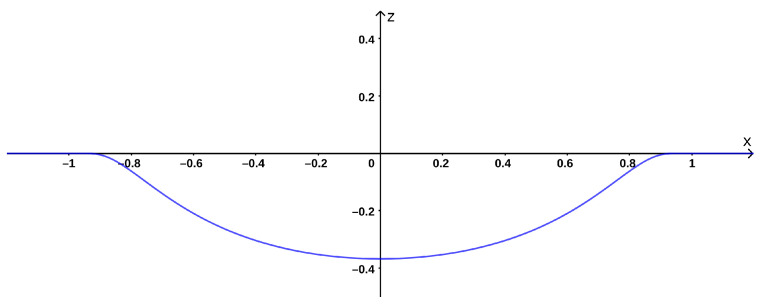
Section of the reference dent function. Maximum depth is 0.368 and just 0.005 at x=0.9.

In the absence of a formal shape definition, the following was chosen as representative *dent function*:

(18) $$z=\left\{\begin{array}{cc} -e^{-\frac{1}{1-r^{2}}} & \mathrm{if}\;|r|<1\\ 0 & \mathrm{elsewhere} \end{array}\right.$$

where $r=\sqrt{x^{2}+y^{2}}$. As for real dents, the function presents a smooth trend and vanishing boundaries (Figure 8). It can be rescaled and rotated to resemble different dents.

**Figure 9:**
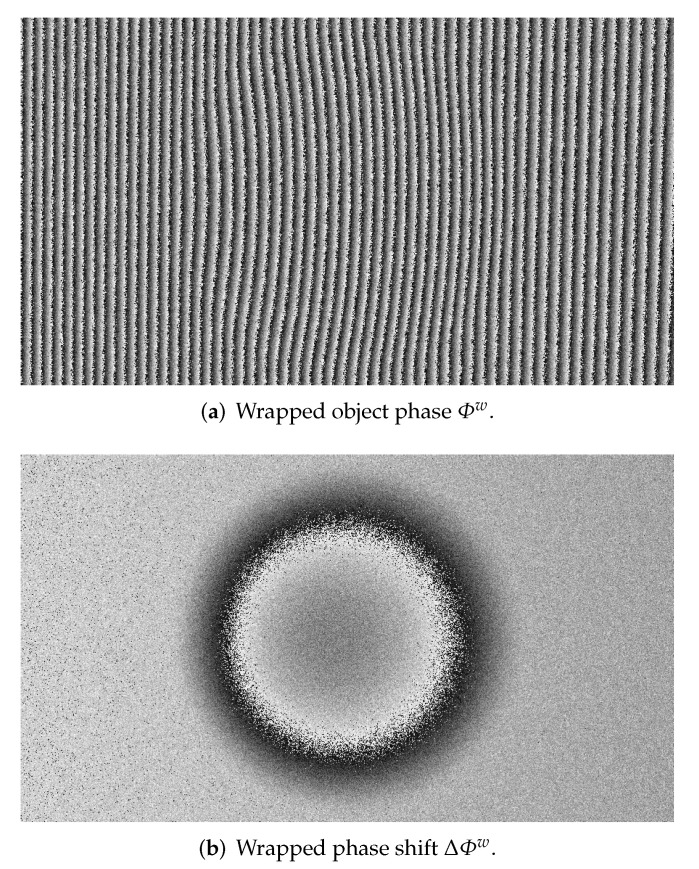
Compared to the (**a**) object phase $\Phi^{w}$, the slower trend of the (**b**) phase shift $\Delta \Phi^{w}$ is clearly visible in a simulated dent with added white noise (σ=0.6).

**Figure 10:**
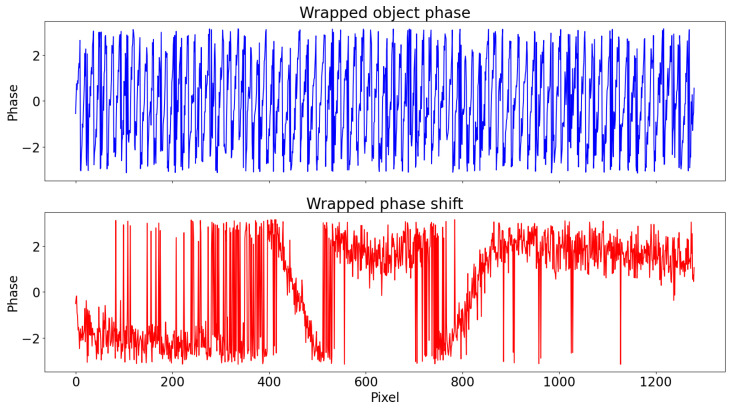
(**Top**) Wrapped phase $\Phi^{w}$ and (**Bottom**) phase shift $\Delta \Phi^{w}$ at middle row. Unwrapping of $\Phi^{w}$ has to manage many more 2π-jumps.

A simulated dent with maximum depth 3 mm and width 30 mm was scanned from 1 m of distance with a resolution of 1280×720 pixels and $T_{p}=8$. White noise (σ=0.6) was added, similarly to Section 2.4. The object phase maps and the middle row signals are shown in Figure 9 and Figure 10, respectively.

**Figure 11:**
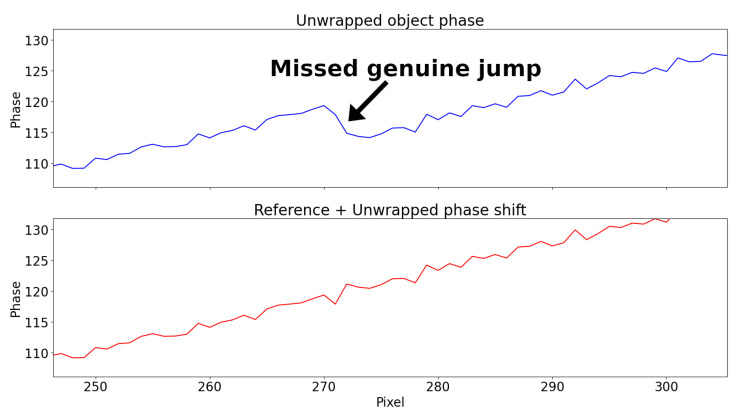
Unwrapping of the simulated dent signal with added white noise (σ=0.6) using Itoh’s algorithm. Detail of one of the several genuine 2π-jumps missed using Equation (11) (**top**) but not using Equation (13) (**bottom**).

Due to noise, Itoh’s unwrapping algorithm missed 2π-jumps on more occasions when using Equation (11) compared to Equation (13). In Figure 11, the detail of a missed genuine 2π-jump is showed.

**Figure 12:**
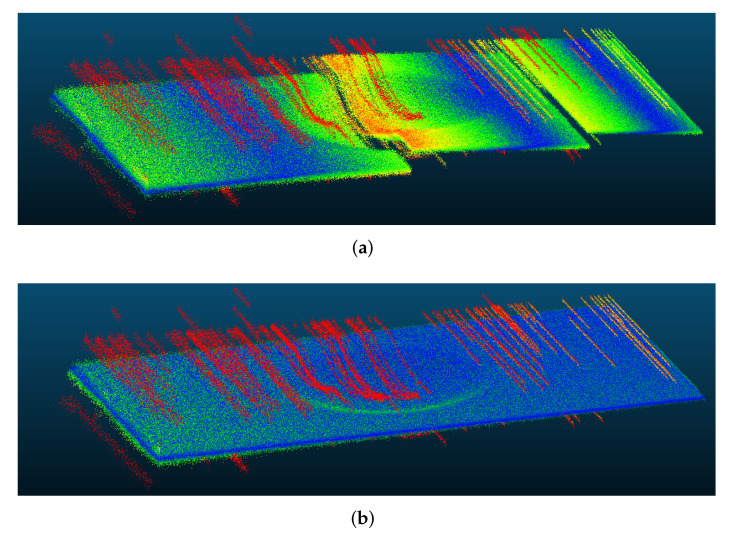
Comparison of 3D reconstruction of the simulated dent (as heatmap of MAE) using Itoh’s algorithm with the (**a**) direct mapping and the (**b**) virtual reference methods.

The mean absolute error (MAE) was chosen as a metric, calculated as the distance between the expected mesh to the point cloud [42]. After 3D reconstruction, the MAE from the original simulated dent was 6.820 mm (its standard deviation was σ=7.174 mm) with the direct method, and 3.827 mm (σ=7.090 mm) with the proposed method. Reconstructed 3D points are shown in Figure 12.

The simulation was repeated using the noise-robust unwrapping algorithm by Estrada et al. [43]. With τ=0.8, the MAE from the original 3D dent was 7.597 mm (σ=4.822 mm) with the direct method and 1.591 mm (σ=1.339 mm) with the proposed method.

**Figure 13:**
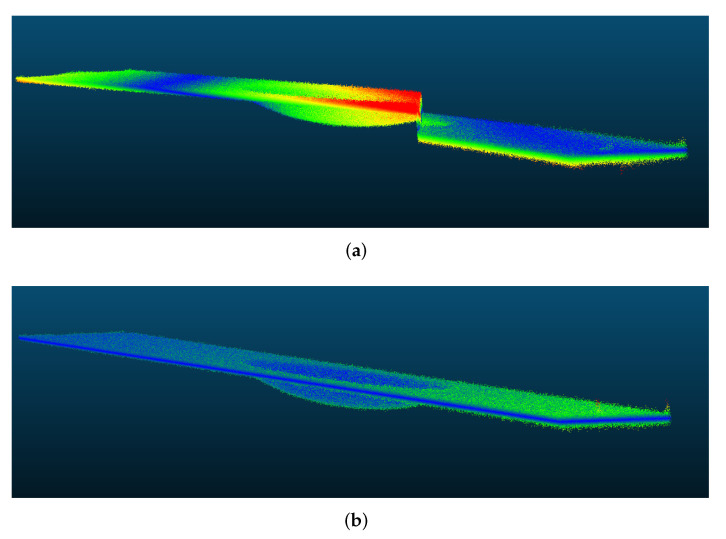
Comparison of 3D reconstruction of the simulated dent (as heatmap of MAE) using Estrada’s noise-robust algorithm (τ=0.8) with the (**a**) direct mapping method and the (**b**) proposed virtual reference method.

In this case, the unwrapping was also more effective using the virtual reference. As shown in Figure 13, in the first case, Estrada’s algorithm is unable to correctly unwrap the phase, leaving a visible artefact. This does not happen with the proposed method due to a reduced number of 2π-jumps to deal with.

Decreasing to τ=0.7 for higher noise suppression (or *regularisation*), the reconstruction was free of artefacts in both methods at the price of lower *dynamic range* [43]. The MAEs were 1.241 mm (σ=1.047 mm) and 1.221 mm (σ=1.032 mm), respectively. Although less evident, the improvement of a virtual reference still applies.

While using Estrada’s noise-robust unwrapping algorithm results in a better reconstruction over Itoh’s algorithm, the proposed virtual reference method visibly improves the final result *independently* from the choice of unwrapping algorithm.

The average running time for the Python implementation was 0.36 s for the direct method and 1.18 s for the proposed method, higher due to the reference image generation and processing.

## 4. Experiments

### 4.1. Static Scenario

An inexpensive and lightweight experimental setup was composed of an ELP USB camera (resolution of 1920×1080 pixels at 30 FPS) and an AnyBeam class 1 laser projector (resolution of 1280×720 pixels). To correct lens distortion, three coefficients for the radial distortion and two for the tangential one were used. The system was calibrated using a method similar to [36] and the resulting baseline was b=235.040 mm. Although not indicative of the accuracy over arbitrary objects [38], Table 1 shows the RMS reprojection errors.

**Figure 14:**
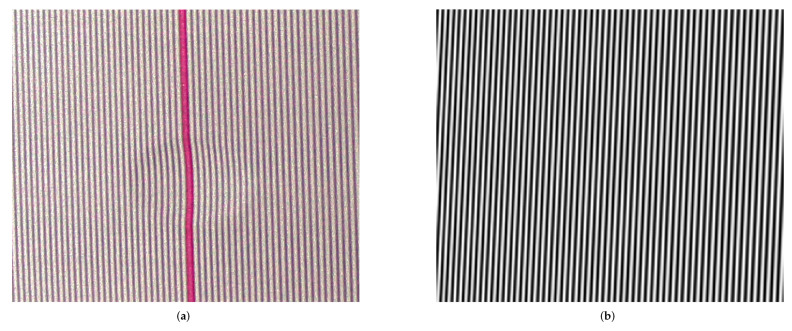
Input images for sample A. (**a**) Captured object image. Red values are converted to greyscale before FFT. (**b**) Virtual reference image.

Following the dent function defined above, four different dented surface samples were modeled and 3D printed as 15 cm × 15 cm tiles with a white matt finish. The samples were scanned from a distance of circa 60 cm using $T_{p}=8$. Images were cropped to 760×640 pixels to select only the tile and remove the background. The characteristics of the samples are reported in Table 2. Captured image and calculated virtual reference plane for sample A are showed in Figure 14.

**Figure 15:**
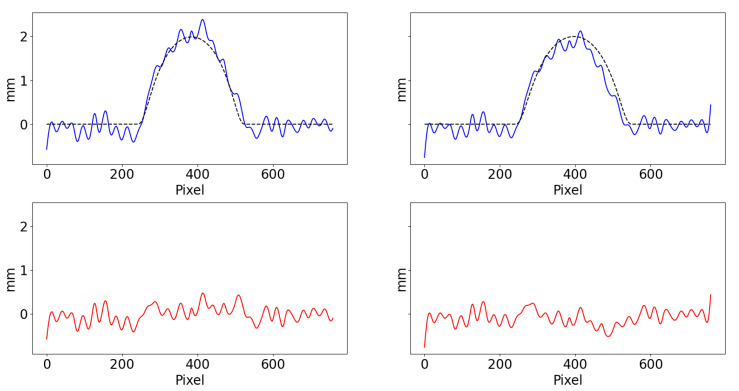
Calculated (solid) and ground truth (dashed) depth of sample A (**top**) and error (**bottom**) along the middle section using direct mapping (**left**) and proposed virtual reference method (**right**). Overall MAE was 0.158 mm and 0.126 mm, respectively.

Figure 15 reports the depth and its error along the middle row of sample A. The causes of the shown noise distribution include reflections, laser speckle, sampling, spectral leakage, border artefacts in the Fourier transform, and systematic error of the 3D printing process.

The MAE and its standard deviation are reported for each sample, comparing the direct mapping and the virtual reference methods using 2D Itoh’s (Table 3) and Estrada’s (Table 4) unwrapping algorithms.

**Figure 16:**
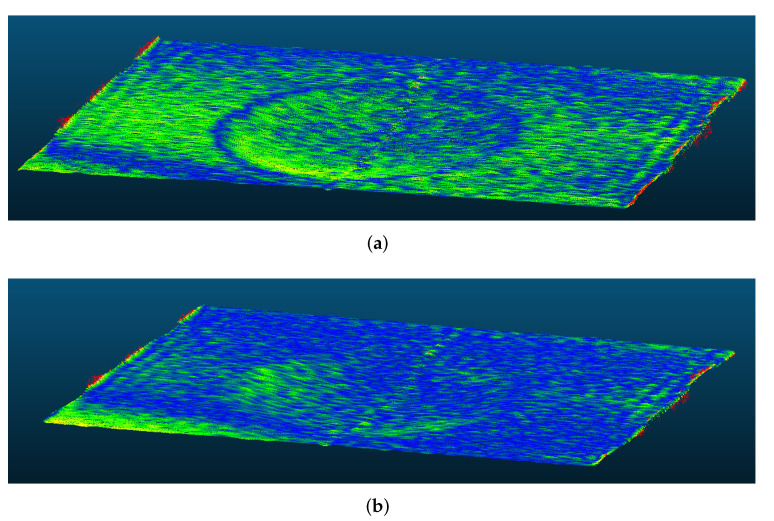
Comparison of 3D reconstruction for sample C (as heatmap of MAE) using Estrada’s unwrapping algorithm with the (**a**) direct mapping and the (**b**) proposed virtual reference methods. Blue areas correspond to lower error than green ones. The border artefacts of the Fourier transform are visible as high-error areas (yellow and red).

Independently from the unwrapping algorithm, the proposed method can generally deliver more accurate 3D reconstructions. The difference is visible, for example, in Figure 16, showing the sample C reconstruction using Estrada’s unwrapping. Border artefacts were responsible for the most of the outliers, accounting for approximately 10% of MAE. In future, the windowed FTP or the wavelet transform could be considered to reduce these errors.

### 4.2. Dynamic Scenario

The class 1 laser projector employed above has the advantage of being focus-free, that is, the projected image is always *in focus* at any distance from the object. This technology is made possible by the use of microelectromechanical systems (MEMS) that drive laser beams with a certain refresh rate, independent from camera exposure time.

This also causes a flickering effect in the camera image, that in the experiments above was compensated by setting high exposure times. Consequently, such a system cannot be used to scan moving objects as is. To maintain the focus-free benefit, the best solution would be to employ a synchronised hardware, so that the camera exposure time corresponds to one projector refresh cycle.

Unfortunately, such technology was not available during this work, thus the projector was replaced with a common LCD one of the same resolution, and the system was recalibrated to test the algorithm over moving objects. Table 5 shows the RMS reprojection errors in this second configuration (b=266.784 mm).

**Figure 17:**
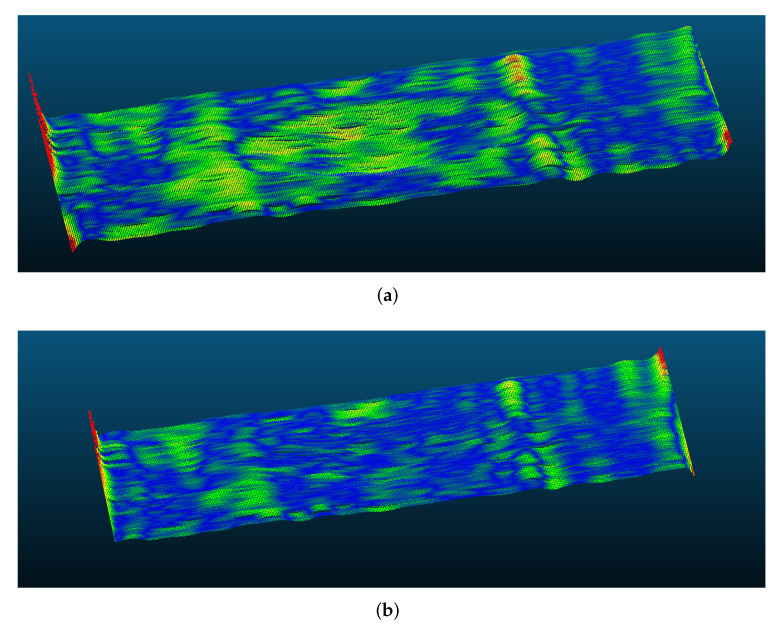
Comparison of 3D reconstruction for sample C acquired during movement using Estrada’s unwrapping algorithm with the (**a**) direct mapping and the (**b**) proposed virtual reference methods. Blue areas correspond to lower error than green ones. The border artefacts of the Fourier transform are visible as high-error areas (yellow and red).

Samples were scanned while moving at a speed of about 0.1 m/s along the *x*-axis at a distance of circa 120 cm. As before, compared results between the two methods are reported in Table 6. This time, only Estrada’s unwrapping algorithm was used, and, for reference, all the values from dynamic scenes were compared with the corresponding static ones of the current setup, to highlight the effect of movement. Figure 17 shows the results for sample C.

As expected, dynamic MAE does not consistently increase when compared with the static one, as the single-shot acquisition is not significantly affected by movement and vibration, provided that motion blur is suppressed by a short exposure time with respect to the speed of the object. MAE is much more affected by other factors, such as object albedo and distance, which are independent of movement.

Overall, if compared with the first configuration, the reduced performance is due to the change of projector and the increased distance. In addition, for this setup, the use of the phase shift, calculated with respect to the virtual reference, performed better than the direct mapping in all the cases. On average, the MAE difference was 0.138 mm, even more relevant than in the first configuration.

## 5. Discussion

To date, most of the UAV-based systems developed for aircraft inspections make use of a monocular camera as a primary acquisition device and are thus incapable of detecting shallow dents or collecting measures [11]. As the acquisition of measures is essential for damage evaluation [7], 3D scanning must be considered for proper automation. However, the employment of 3D scanning technology on UAVs is not straightforward: high-accuracy 3D scanners are generally based on multiple-shot algorithms, as the projection of many different patterns allows for detailed codification, and are incompatible with fast movements and vibration that would be experienced while scanning from a flying drone.

This work showed that the FTP should be considered to automate aircraft dent inspections. The single-shot nature of the method, together with its high phase sensitivity to depth variations, constitutes an appropriate solution for the integration with UAVs, ultimately providing fast and effective inspections. Table 7 summarises existing dent inspection approaches, comparing their pros and cons against the proposed one.

FTP is naturally resilient to movement and vibration, although codification information must be condensed into a single pattern, implying local smoothness. While the local smoothness assumption is true for most of the aircraft skin, the major challenge for effective application in the field is the increase of signal-to-noise ratio, especially from longer distances. This translates into overcoming surface albedo and employing a projection technology capable of operating in common indoor or even outdoor light conditions, generally not dark enough.

The use of light sources outside the visible spectrum or higher class lasers might be considered. However, while the use of a laser projector highly improves the contrast of the projected pattern, it normally introduces the speckle phenomenon, presented as a granular pattern over the camera image that affects the filtered phase and, consequently, produces noise in the final measures. Speckle can be corrected with different types of *despecklers* [44].

## 6. Conclusions

The aviation industry is increasingly making use of UAVs for aircraft inspections. To date, a UAV-based solution for the detection and measurement of dents has yet to be found, as high-accuracy 3D scanners usually work with multiple-shot algorithms and are thus incompatible with fast movements and vibration.

Triangular stereo Fourier transform profilometry (FTP) was proposed here to evaluate its potential towards automated aircraft dent inspections. Additionally, two modifications to the FTP algorithm were introduced. First, the automatic band-pass estimation was presented via a *virtual* reference plane, thus eliminating the user interaction in the filtering process. Secondly, after showing that the interaction of noise over 2π-jumps is lower using the phase shift, a *virtual* reference image was employed to increase accuracy.

Overall, the proposed method was able to faithfully reconstruct the 3D point clouds of demonstrative dents at distances of 60 cm and 120 cm, delivering sub-millimeter accuracy despite the use of unsophisticated hardware. Simulations and experiments with 3D-printed dent samples showed that the employment of the virtual reference delivers better results, independent of the choice of unwrapping algorithm.

With additional research, an FTP-based system could be integrated with a UAV, enabling quick and effective dent inspection. Solutions to deal with light conditions in common operational environments are to be found. It must also be noted that a successful 3D data acquisition is just the first step for comprehensive automation, as the difficulties in detecting dents in the physical world are somehow translated into the digital world. As such, an end-to-end automatic system for dent detection and measurement remains an open challenge in MRO. 

## Figures and Tables

**Table 1:** RMS reprojection errors of the first configuration.

Camera	Projector	Stereo
0.477	1.231	2.686

**Table 2:** Dimensions of dent samples.

Sample	Length (mm)	Width (mm)	Depth (mm)
A	60	40	2
B	120	100	2
C	100	80	3
D	120	80	1

**Table 3:** Compared MAE (and standard deviation) of the direct mapping versus the proposed virtual reference method with 2D Itoh’s unwrapping algorithm. The last column reports the absolute and percentage difference.

	Direct Mapping	Proposed	Difference
Sample	MAE (std) in mm	MAE (std) in mm	mm (%)
A	0.158 (0.176)	**0.126** (0.115)	0.032 (22.5%)
B	0.167 (0.202)	**0.144** (0.151)	0.023 (10.1%)
C	0.259 (0.616)	**0.154** (0.494)	0.105 (50.8%)
D	0.158 (0.566)	**0.144** (0.537)	0.014 (9.3%)

**Table 4:** Compared MAE (and standard deviation) of the direct mapping versus the proposed virtual reference method with Estrada’s unwrapping algorithm (τ=0.8). The last column reports the absolute and percentage difference.

	Direct Mapping	Proposed	Difference
Sample	MAE (std) in mm	MAE (std) in mm	mm (%)
A	0.153 (0.174)	**0.120** (0.110)	0.033 (24.2%)
B	0.165 (0.190)	**0.135** (0.130)	0.030 (20.0%)
C	0.202 (0.162)	**0.115** (0.114)	0.087 (54.9%)
D	0.213 (0.368)	**0.124** (0.131)	0.089 (52.8%)

**Table 5:** RMS reprojection error of the second configuration.

Camera	Projector	Stereo
0.282	0.772	2.625

**Table 6:** Compared MAE (and standard deviation) of the direct mapping versus the proposed virtual reference method with Estrada’s unwrapping algorithm (τ=0.8) over both static and dynamic samples.

		Direct Mapping	Proposed
Sample	Type	MAE (std) in mm	MAE (std) in mm
A	Dynamic	0.381 (0.456)	**0.280** (0.307)
	Static	0.504 (0.486)	**0.285** (0.217)
B	Dynamic	0.419 (0.438)	**0.309** (0.396)
	Static	0.432 (0.329)	**0.287** (0.255)
C	Dynamic	0.436 (0.443)	**0.271** (0.265)
	Static	0.493 (0.546)	**0.320** (0.468)
D	Dynamic	0.411 (0.394)	**0.295** (0.294)
	Static	0.370 (0.357)	**0.293** (0.401)

**Table 7:** Detection and measures of dents in MRO. Summary of pros and cons for different inspection approaches.

Approach	Pros	Cons
Traditional	No special hardware requirements Straightforward	Hazardous Subjective output Time-consuming
Handheld 3D scanner	High accuracy Repeatable	Hazardous Time-consuming
Monocular camera UAV	Fast Repeatable Safe	No measures Shallow dents not detectable
UAV equipped with FTP	Fast Repeatable Safe Measures collected	Complex light control Smoothness assumption
